# The Future of St. Bartholomew's Hospital

**Published:** 1903-11-21

**Authors:** Philip J. Hensley

**Affiliations:** Consulting Physician to St. Bartholomew's Hospital.


### EDITOR'S LETTER-BOX.

[Our Correspondents are reminded that prolixity is a great bar to publication, and that brevity of style and conciseness of statement greatly facilitate early insertion.]

### THE FUTURE OF ST. BARTHOLOMEW'S HOSPITAL.

Sir,—I should be obliged if you would allow me to supply corrections to certain statements contained in a letter published in your last issue headed "The Future of St. Bartholomew's Hospital" by Mr. Motion, a Governor of the hospital.

I may say to begin with that I sympathise most heartily with what I understand to be Mr. Motion's ultimate aim; so much so that at the meeting of the Governors on Thursday, November 5th, I was strongly minded, though it was the first occasion on which I had sat as a Governor, to second and support his resolution.

I refrained from doing so because it seemed to me that he had not succeeded in presenting matters in that happy manner which might lead to a good result, and that the only effect might be to postpone the beginning of movement towards the desired end.

According to Mr. Motion there are two rival policies—one which he speaks of as the official scheme or the Mansion House plan, which he says will merely put a modern gloss on old faults and provide a hospital with grave and essential defects; another or alternative scheme, apparently the child of "Criticism," which, he says, if adopted, will secure everything that modern experts advise, and will, at the same time, guarantee the opportunity for such developments as are probable in the future.

This does not, in my opinion, in the least represent the actual position, which, as I understand it, is simply this: Plans for new casualty and out-patient departments have been approved, and it has been decided that buildings, according to these plans, subject to any modifications as from time to time may appear desirable, shall at once be commenced.

This, it would seem, is all that is really settled; it is a good beginning which provides for what, after careful consideration, has been recognised to be most urgently required. There does not seem in it anything which need prevent future development, or hamper further plans for the rest of the hospital, or any reason to fear that this need be unsatisfactorily provided, adequate space be allowed, and ample funds be forthcoming.

It is, I believe, a fact that the late Lord Mayor set his face altogether against the acquirement of more ground, and by taking that attitude no doubt modified the potentiality of a Mansion House appeal, at any rate in his time. But there is now a new Lord Mayor; and even all mayors are not cut according to the same pattern, made of the same stuff, or moved by the same feelings.

Thus while we may estimate Mr. Motion's picture of the present as misleading, we may hope that he may not be justified in his doubts as to the future.

It is of minor importance to point out his inaccuracy as to the past.

He speaks of the "silence of the staff." All I can say as to this is, that if there has been the appearance of silence, it is wholly and solely to be attributed to the deafness of those whose business it should have been to have heard.

For years past has the staff been sending in to those in authority reports pointing out the requirements of the hospital and indicating desiderata towards its future development.

To what extent these documents have reached the general body of Governors I do not know; but I do know that on August 22nd, 1901, a copy of a certain most important report was forwarded by the staff to every Governor, and if Mr. Motion was a Governor at that time I cannot understand his writing as he does in the letter you published last week.

Personally I feel particularly aggrieved by this accusation of dumbness, having been in fact less dumb than it is my nature to be, inasmuch as on one occasion I took the unusual course of sending in a special paper supplementary to what was sent by my colleagues.

The aim of this was mainly to give my views as to position of new ward blocks, and it pretty clearly indicated how very desirable it was that there should be extension of space to the south.

While it seems hard thus to be accounted dumb in the past, I have also lately received from the hospital a hint, in the shape of a long green wand, that I must consider myself lame and no longer fit for its service; and now having become a Governor I shall no doubt for the future among my late colleagues have the credit of being ex officio deaf.

Yours,

Philip J. Hensley,

(Consulting Physician to St. Bartholomew's Hospital.)

⁂ Dr. Hensley cannot have studied Plan No. 6, which places the new out-patient department behind the present medical school buildings. It is to occupy such a portion of the new land already purchased that whenever it is built it will be impossible to plan a modern hospital on the site should it be enlarged to 8½ acres. Dr. Hensley thus affords evidence that he has failed to study the site as a whole, and his argument rests, therefore, upon a misapprehension of the factors in the problem to be solved. No doubt the Mansion House Committee and the Governors are suffering from the same failure to apprehend both the possibilities and requirements of the present site of St. Bartholomew's Hospital. It follows that no steps should be taken to commence the new out-patient buildings under Plan No. 6 until the questions of an 8½-acre site and a new hospital are finally determined. —Ed. The Hospital.

